# Sexual experiences of women during and after pregnancy: a survey-based study

**DOI:** 10.1186/s12884-026-09648-3

**Published:** 2026-07-14

**Authors:** G. Del Bravo, A. Ferrari, M. Giudetti, V. Valentino, M. M. Montt-Guevara, T. Simoncini, P. Mannella

**Affiliations:** 1 Department of Clinical and Experimental Medicine, Division of Obstetrics and Gynaecology, University of Pisa, Via Roma 67, Pisa, 56126 Italy; 2 Scuola Superiore Sant’Anna, Institute of Management, MeS (Management and Health) Laboratory, Pisa, Italy

**Keywords:** Pregnancy, Postpartum, Sex, Survey, Couple, Relationship

## Abstract

**Background:**

Pregnancy and postpartum are critical phases with major physical, psychological, and relational changes. Sexuality, though central to well-being, is underexplored in clinical practice and clouded by social taboos. Literature mainly focuses on dysfunctions, overlooking the interplay of psychological and relational factors shaping women’s sexual satisfaction. Little is known about why women may engage in sex to satisfy their partner. We investigated self-reported sexual health experiences during pregnancy and postpartum, focusing on psychological, relational, and physical factors. We explored factors associated with reporting intercourse for a partner’s pleasure.

**Methods:**

A cross-sectional online survey was conducted in Italy (September 2024-March 2025). Women ≥ 18, pregnant or within six months postpartum, anonymously completed a questionnaire on six domains: sexual activity and desire, relationship dynamics, physical and psychological changes, fears and difficulties, the gynecologist’s role, and puerperium. The primary outcome was having sex for the partner’s pleasure. Multivariate logistic regression was used to explore the associations with the primary outcome.

**Results:**

Among 629 respondents (246 pregnant, 383 postpartum), sexual behavior changed markedly across pregnancy and postpartum. During pregnancy, 56% of women in the first trimester and 50% in the third reported sex only to satisfy their partner, rising to 69% postpartum. Pain during intercourse was reported by 28% in the first/second trimester and 45% in the third, while fear of vaginal penetration increased from 60% to 75%. Postpartum, 47% reported difficulty in sexual intercourse within two months of delivery, rising to 63% within six months. Having sex for the partner’s pleasure was associated to being postpartum (OR 1.9) and reporting decreased physical energy (OR 2.0). Among pregnant women, dyspareunia and fear of jeopardizing the relationship were significant predictors; after childbirth, resuming sex within two months was associated with the same outcome.

**Conclusion:**

Findings highlight a gap in perinatal care, where sexual health is often neglected. Integrating counseling into routine obstetric care, and addressing fatigue, pain, body image, and partner dynamics, could foster healthier and more autonomous sexual experiences. Proactive counseling and greater clinical attention to fatigue, pain, and relational dynamics are needed to promote women’s sexual well-being perinatally.

**Supplementary Information:**

The online version contains supplementary material available at https://doi.org/10.1186/s12884-026-09648-3.

## Background

Pregnancy and puerperium are key phases in women’s lives characterized by significant physical, hormonal, emotional, and relational changes. The current literature confirms that sexual satisfaction during these periods is an important aspect in women’s lives that tends to be neglected and treated with reticence [1].

As defined by the World Health Organization, health is not simply the absence of disease or pathological physical and mental dysfunction, but a “state of physical, emotional, mental and social well-being” [2]. Thus, sexual health can be considered as an indicator of overall health in pregnant and postpartum women, while sexual dysfunction and dissatisfaction as indicators of morbidity.

Discussion on this topic is often limited by cultural taboos, lack of adequate sex education, and stigmatization. In fact, the perception and experience of sexuality vary from individual to individual and are deeply intertwined with the culture to which they belong, contributing to its complexity [3]. Although the topic of sexuality in pregnancy and postpartum may be a source of concern and discomfort, women may not seek advice and help from health professionals, particularly the gynecologist.

In addition, several factors can influence sexual function and well-being during these delicate phases of women’s lives, such as pregnancy-induced hormonal and physical changes, the perception of a new body image, the experience of childbirth (both personal and shared with the partner), possible perineal tears during childbirth, breastfeeding, and newborn care [4, 5].

Numerous studies on sexuality during pregnancy and after childbirth have been published, and most of them have analyzed sexual dysfunction in pregnancy and postpartum using validated generic questionnaires such as the Female Sexual Function Index (FSFI). In more recent studies, further attention has also been paid to the psychological and relational component of the couple, as well as the beliefs and fears that women feel and often do not talk about [6–8].

This survey-based study aims to investigate sexual experiences of Italian women during pregnancy and postpartum, highlighting the psychological and relational aspects of the couple, and unveiling difficulties and fears encountered. Attention was also given to the role of health professionals, particularly the gynecologist, who most frequently cares for women during these life phases. The goal is to contribute to a greater awareness among health professionals, couples and society, so that a more serene, unprejudiced, and informed approach toward sexuality during these periods of great transformations can be fostered.

## Methods

### Study setting, study design, and participants

This was a cross-sectional survey-based study carried out in Italy. In Italy, healthcare is provided through a universal public health system (Servizio Sanitario Nazionale), which ensures access to essential medical services for all residents, including maternity and reproductive health care, largely free of charge at the point of delivery. Obstetric and gynecological services are generally delivered through a network of public hospitals and territorial clinics, with standardized prenatal and postpartum care protocols regulated at the national level.

The authors developed a questionnaire in Italian on sexual well-being during pregnancy and postpartum using Google Forms (Appendix A1). To build the questionnaire, a literature review was performed, from which no already validated questionnaires or scales emerged. As a matter of facts, the lack of specific questionnaires that thoroughly investigated the psychological and relational sphere of pregnant / postpartum women prompted us to create a new questionnaire with newly developed questions. Although the questionnaire was developed based on a literature review, it was not formally validated, and its psychometric properties were not assessed. The questionnaire was anonymous to encourage honest reporting and reduce social desirability bias, given the sensitive nature of the topic.

Before distributing the study, we conducted a pilot test of the questionnaire with a convenience sample of 40 women to assess its clarity and comprehensibility using a 1-to-5 rating scale. However, no formal psychometric validation was performed. A total of 93% of participants rated the questionnaire as *well* (4/5) or *completely understandable* (5/5). Participants were also invited to identify any sections they found difficult to interpret. Finally, we asked whether they considered any of the questions unnecessary and if they wished to suggest additional items that had not been included.

The final version of the questionnaire was disseminated from September 2024 to March 2025. Women were recruited thanks to an online campaign conducted through social media to disseminate the questionnaire link nationwide. Inclusion criteria were age over 18 years, pregnancy or postpartum within 6 months after delivery. No exclusion criteria related to clinical and psychiatric conditions or high-risk pregnancies were applied. Due to anonymous dissemination via social media, it was not possible to track the number of individuals reached, link views, or participation rates. Women were characterized by age and parity.

The questions were divided according to the time of response: pregnancy (first, second, third trimester) vs. postpartum (within 2 months after delivery, between 2 and 6 months). Questions were also grouped into six areas of interest: sexual desire and intercourse, couple relationship, physical and psychological changes in women, difficulties and fears, role of the gynecologist, and puerperium.

### Statistical analysis

Three study populations were considered: (1) all respondents, (2) women who responded during pregnancy, and (3) women who responded during postpartum. For each population, we reported women’s answers (categorical variables) as counts and percentages by response time (first trimester, second trimester, third trimester, < 2 months postpartum, 2–6 months postpartum).

We used as the primary outcome of the study the question “Have you had sexual intercourse only to satisfy your partner’s pleasure (during or after pregnancy)?” (Yes/No). For each study population (total population, pregnancy population, and postpartum population), we performed multivariate logistic regression models to explore the association of the outcome variable with all other responses to the questionnaire. We chose a single-item dichotomous question as the primary outcome to ensure clarity and feasibility in a web-based survey, even though this approach may not fully capture the multidimensional nature of sexual experiences. This outcome was selected as a pragmatic indicator of potential sexual dissatisfaction and relational imbalance in the perinatal context.

Considering only the population of women who responded during pregnancy, we considered as the secondary outcome the question “Have you experienced difficulty or pain during sexual intercourse during pregnancy?” (Yes/No). As for postpartum responders, we selected as the secondary outcome the question “Have you had sexual intercourse within two months after delivery?” (Yes/No).

Data management was performed in SAS Software (SAS Institute Inc., Cary, NC, USA) version 9.4, while statistical analyses on Stata Software version 17.0 (Stata-Corp, LLC, College Station, Texas, USA). Statistical significance was set at a p-value less than 0.05.

## Results

We obtained a population of 629 respondents. About half of the women were 30–34 years old, with women who responded in the postpartum being significantly older (Table 1). Approximately 70% of women were primigravida. Women reporting that they had sex only to satisfy their partner’s pleasure accounted for 56% in the first trimester and 50% in the third trimester of pregnancy (Table A1); this proportion rose to 69% in the postpartum period (Table 1). About 73% of respondents reported a decrease in physical energy in the first and third trimesters of pregnancy, while only 66% during the second trimester (Table A1); this figure reached 81% postpartum (Table 1). Only 18% of women had counselling with their gynecologist about sexuality, although 24% of women had sought advice from their gynecologist. Counseling with the gynecologist was mainly carried out during the first trimester of pregnancy, while women sought advice from the gynecologist mainly after childbirth (Table 1 and Table A1).

**Table 1:** Sociodemographic and clinical characteristics of participants during pregnancy and postpartum (group differences were evaluated using the chi-squared test, with p-values < 0.05 indicating statistical significance)

Number	Pregnancy	Postpartum	Total	*p*-value
	246	383	629	
Age class				0.018
<30	61 (24.8%)	71 (18.5%)	132 (21%)	
30–34	119 (48.4%)	181 (47.3%)	300 (47.7%)	
35–39	58 (23.6%)	97 (25.3%)	155 (24.6%)	
>39	8 (3.3%)	34 (8.9%)	42 (6.7%)	
Parity				0.53
Primigravida	171 (69.5%)	257 (67.1%)	428 (68%)	
Multiparous	75 (30.5%)	126 (32.9%)	201 (32%)	
Sex for man’s pleasure				< 0.001
No	117 (47.6%)	120 (31.3%)	237 (37.7%)	
Yes	129 (52.4%)	263 (68.7%)	392 (62.3%)	
Decreased energy				0.004
No	72 (29.3%)	74 (19.3%)	146 (23.2%)	
Yes	174 (70.7%)	309 (80.7%)	483 (76.8%)	
Counselling with ObGyn on sex				0.62
No	203 (82.5%)	310 (80.9%)	513 (81.6%)	
Yes	43 (17.5%)	73 (19.1%)	116 (18.4%)	
Advice from ObGyn on sex				< 0.001
No	205 (83.3%)	273 (71.3%)	478 (76%)	
Sought	41 (16.7%)	110 (28.7%)	151 (24%)	

**Fig. 1:**
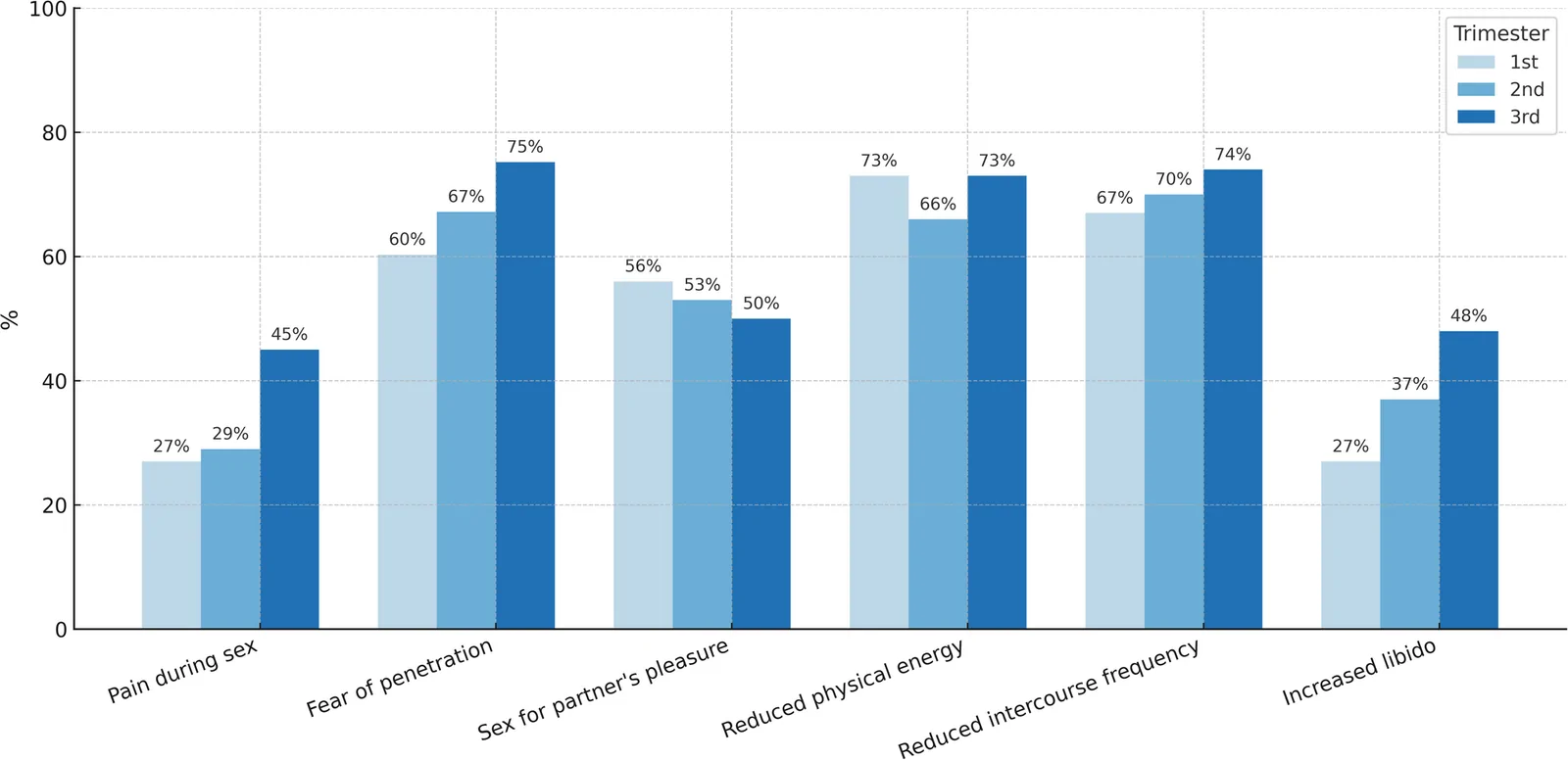
Selected sexual health responses of women during pregnancy by trimester

**Fig. 2:**
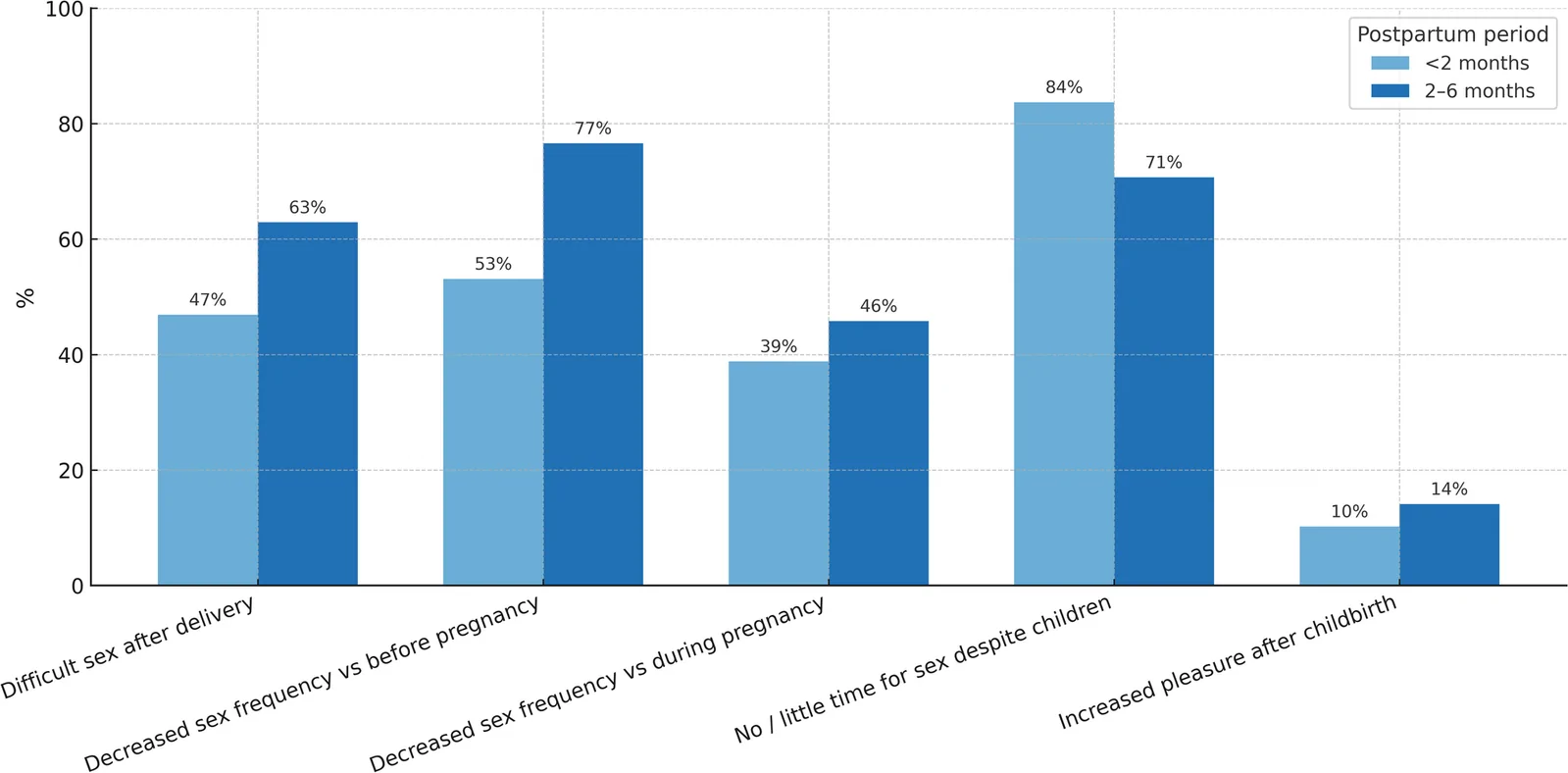
Selected sexual health responses of women after childbirth by trimester

The number of women who answered the questionnaire during pregnancy was 246, while 383 women responded to the questionnaire after childbirth (Table A1 and Table A2). During pregnancy, 28% of women in the first/second trimester and 45% in the third trimester reported pain during intercourse, while 60% and 75% of women reported fear of vaginal penetration in the first and third trimester, respectively (Fig. 1). Within two months postpartum, 47% of women reported difficulty in sexual intercourse; this proportion rose to 63% within six months postpartum (Fig. 2). Please, see Table A1 and Table A2 of the Appendices for further details on the responses to the pregnancy and postpartum questionnaires by the two study sub-populations.

In the entire study population (Table 2), the factors associated to having sex only for partner satisfaction were postpartum (OR 1.9, 95% CI 1.4 to 2.7) and decreased physical energy (OR 2.0, 95% CI 1.4 to 3.0). For the sub-population of women who responded during pregnancy (Table 3), the factors associated to having sex only for the partner’s pleasure were difficulty in sexual intercourse because of pain (OR 2.1, 95% CI 1.1 to 3.8) and being afraid of jeopardizing the relationship with the partner sometimes (OR 2.1, 95% CI 1.1 to 4.1) or always (OR 2.9, 95% CI 1.0 to 8.2). For the sub-population of women who responded during postpartum (Table 4), decreased energy was confirmed to be significantly associated to the primary outcome (OR 3.0, 95% CI 1.6 to 5.4); moreover, women who had sex within the first two months after delivery were more likely to do so only for the partner’s pleasure (OR 1.8, 95% CI 1.0 to 3.0).

**Table 2:** Logistic regression model for the primary outcome in the entire study population. Total study population (*n* = 629)

Sex for the partner’s pleasure	OR	*p*-value	95% CI
Postpartum vs. pregnancy	1.9	< 0.001	1.4 to 2.7
30–34 vs. < 30	0.9	0.773	0.6 to 1.5
35–39 vs. < 30	0.8	0.459	0.5 to 1.4
> 39 vs. < 30	0.9	0.760	0.4 to 1.9
Multiparous vs. primigravida	1.1	0.645	0.8 to 1.6
Decreased energy vs. not	2.0	< 0.001	1.4 to 3
Counselling with ObGyn on sex vs. not	1.1	0.622	0.7 to 1.8
Advice from with ObGyn sought vs. not	0.8	0.419	0.6 to 1.3

*OR* Odds Ratio, *CI* Confidence interval, *ObGyn* Obstetrician-Gynecologist

**Table 3:** Logistic regression model for the primary outcome in the pregnancy sub-population. Pregnancy sub-population (*n* = 241)

Sex for the partner’s pleasure	OR	*p*-value	95% CI
2nd vs. 1st trimester	0.8	0.597	0.4 to 1.8
3rd vs. 1st trimester	0.7	0.422	0.4 to 1.5
Increased libido during pregnancy	0.6	0.122	0.3 to 1.2
30–34 vs. < 30	0.8	0.516	0.4 to 1.6
35–39 vs. < 30	0.6	0.300	0.3 to 1.5
> 39 vs. < 30	2.1	0.387	0.4 to 11.4
Multiparous vs. primigravida	1.3	0.425	0.7 to 2.4
Decreased energy vs. not	0.9	0.819	0.5 to 1.8
Counselling with ObGyn on sex vs. not	1.0	0.916	0.5 to 2.3
Increased libido while seeking pregnancy	1.3	0.414	0.7 to 2.3
Decreased vs. increased frequency of sex in pregnancy	0.7	0.456	0.3 to 1.8
Not changed vs. increased frequency of sex in pregnancy	0.9	0.901	0.3 to 2.7
Difficult intercourse due to pain	2.1	0.021	1.1 to 3.8
Little vs. no fear for penetration	1.5	0.255	0.7 to 3.1
Great vs. no fear for penetration	1.1	0.781	0.5 to 2.4
Little vs. no importance of orgasm	3.3	0.234	0.5 to 23.2
Great vs. no importance of orgasm	2.9	0.257	0.5 to 17.8
Complete vs. no conversation with partner on sex	0.7	0.308	0.3 to 1.4
Partial vs. no conversation with partner on sex	1.1	0.706	0.6 to 2.2
No / poor emotional connection with partner vs. yes	1.1	0.809	0.5 to 2.7
Neutral vs. comfortable with physical changes in pregnancy	1.0	0.994	0.5 to 2
Uncomfortable vs. comfortable with physical changes in pregnancy	1.0	0.914	0.5 to 1.9
No / poor impact of sexual satisfaction on couple vs. yes	1.0	0.894	0.6 to 1.9
Sometimes vs. never fears for jeopardizing relationship	2.1	0.028	1.1 to 4.1
Always vs. never fears for jeopardizing relationship	2.9	0.047	1 to 8.2

*OR* Odds Ratio, *CI* Confidence interval. *ObGyn* Obstetrician-Gynecologist

**Table 4:** Logistic regression model for the primary outcome in the postpartum sub-population. Postpartum sub-population (*n* = 383)

Sex for the partner’s pleasure	OR	*p*-value	95% CI
2–6 vs. < 2 months postpartum	0.7	0.397	0.3 to 1.6
Increased sexual pleasure postpartum	1.0	0.886	0.5 to 1.9
30–34 vs. < 30	1.0	0.921	0.5 to 1.9
35–39 vs. < 30	1.0	0.969	0.5 to 2.1
> 39 vs. < 30	0.8	0.612	0.3 to 2
Multiparous vs. primigravida	1.1	0.645	0.7 to 1.9
Decreased energy vs. not	3.0	< 0.001	1.6 to 5.4
Counselling with ObGyn on sex vs. not	0.9	0.626	0.5 to 1.6
Great vs. moderate influence of childbirth on couple	0.6	0.197	0.3 to 1.3
No vs. moderate influence of childbirth on couple	0.8	0.560	0.4 to 1.7
Little vs. moderate influence of childbirth on couple	0.7	0.280	0.3 to 1.4
Sex within 2 months postpartum vs. not	1.8	0.036	1 to 3
Decreased vs. increased frequency of sex before pregnancy	1.0	0.996	0.3 to 3.1
Not changed vs. increased frequency of sex before pregnancy	0.5	0.232	0.2 to 1.5
Decreased vs. increased frequency of sex in pregnancy	1.0	0.979	0.5 to 2
Not changed vs. increased frequency of sex in pregnancy	1.2	0.634	0.6 to 2.2
Difficult sex postpartum vs. not	1.4	0.232	0.8 to 2.3
No / little time for sex despite children vs. a lot	1.3	0.317	0.8 to 2.4

As for the secondary outcomes, difficulty or pain during intercourse was more frequently reported by pregnant women during the third trimester of pregnancy than during the first one (OR 3.0, 95% CI 1.3 to 6.6) (Table A3). Also, women who were more likely to report pain or difficulty during intercourse were those who had intercourse only for the partner’s pleasure (OR 2.1, 95% CI 1.1 to 3.9) and those who were not completely comfortable with the physical changes that happened in pregnancy than those who were so (OR 2.5, 95% CI 1.2 to 5.2).

Women who had sex within two months after delivery were more likely to report having sex only to satisfy their partner’s pleasure (OR 1.8, 95% CI 1.1 to 3.1) (Table A4). Women who reported little impact (OR 2.7, 95% CI 1.4 to 5.3) or no impact (OR 2.9, 95% CI 1.4 to 6.0) of the childbirth experience on couple life were more likely to have sex within two months. Finally, women who had little time to have sex due to child-bearing duties had less frequently sex within two months after childbirth (OR 0.4, 95% CI 0.2 to 0.7).

## Discussion

### Main findings

In this survey-based study, we found a reduction in the frequency of sexual intercourse across all three trimesters of pregnancy, particularly during the third trimester (Table A1). We observed that vaginal pain during intercourse and difficulty in complete vaginal intercourse significantly increased as pregnancy progressed, being mostly reported in the third trimester of pregnancy (Table A3). A reduction in the frequency of sexual intercourse was also observed during the postpartum period compared to the pre-conception period (Table A2). Pain during vaginal intercourse was also more frequent among women who were uncomfortable with the para-physiological physical changes of pregnancy (Table A3). Women who reported little to no impact of childbirth on their relationship were more likely to resume sexual intercourse within two months after delivery (Table A4).

We observed that fear of jeopardizing the relationship was significantly associated to engaging in sexual intercourse to satisfy the partner. We also found that decreased physical energy was associated to engaging in sexual intercourse for the partner’s pleasure, both in the general study population and specifically in the postpartum subgroup (Tables 2 and 4). We showed that pregnant women who engaged in sexual intercourse only to satisfy their partner were more likely to experience pain during intercourse, and conversely, women who experienced dyspareunia were more likely to have sex only to please their partner (Table 3 and Table A3). We also observed that women who resumed sexual activity within two months after childbirth were more likely to do so for their partner’s pleasure (Table 4).

These findings should be interpreted as exploratory since our primary outcome was not a unidimensional construct and may encompass multiple dimensions, such as reduced desire, relational negotiation, emotional closeness, and partner-oriented intimacy. Indeed, engaging in sexual intercourse to satisfy the partner may reflect different relational dynamics, including perceived sexual obligation, relational compromise aimed at preserving intimacy, or discrepancies in sexual desire between partners: distinguishing among these dimensions is important for clinical interpretation. In some cases, partner-oriented sexual activity may be associated with reduced desire or relational imbalance, while in others it may derive from relational negotiation, emotional closeness, or efforts to maintain intimacy; therefore, our findings should be treated with caution. Further caution in interpreting results should be paid among pregnant respondents, as the number of predictors relative to events may have reduced estimate stability, increasing the risk of model overfitting.

Our findings are supported by prior studies. Consistently with our work, Von Sydow K and Johnson CE reported a reduction in sexual activity during pregnancy, which may be related to fatigue, back pain, dyspareunia, infections, and vulvar varicosities. They argued that the increased vaginal pain during intercourse in the third trimester of pregnancy may be attributed to a decrease in vaginal connective tissue and a thickening of the muscular fibers of the vaginal canal in preparation for delivery, along with vaginal vasal congestion and reduced lubrication [4, 9]. The reduction in the frequency of sexual intercourse that we observed postpartum compared to the pre-conception period was similarly described in the literature [10]. In line with our results, literature shows that pregnancy-related body shape changes can play a significant role in sexual experience by increasing body dissatisfaction in women [11]. According to Leeman and Rogers, the average time to resume sexual activity was five to six weeks postpartum for more than half of the women [12]; these findings are consistent with our data.

Several studies have highlighted that the childbirth experience, along with fears related to vaginal sutures following perineal trauma, or concerns about physical injury, can reduce the frequency of postpartum sexual activity [9, 13]. These findings, consistently with our study, suggest that the perception of the childbirth experience and concerns about long-term physical consequences may significantly impact postpartum sexuality.

Another aspect that should not be underestimated is the tendency of women during pregnancy and the postpartum period to engage in sexual activity not due to genuine desire, but rather to maintain their relationship. This aspect was noted by us, in line with Jawed-Wessel and Sevick [10]. In certain cultures, there is a deeply rooted belief that sex is a duty of a person who “loves” or is part of a romantic relationship. During these life stages, a woman may feel “obliged” to have sex to conform to social norms, meet traditional partner expectations, or preserve the relationship – despite a potentially reduced sexual desire.

Hipp L. et al. demonstrated in their study that fatigue negatively affects sexual desire and satisfaction [14]. Although our primary outcome (i.e., sexual intercourse for the partner’s pleasure) may not fully reflect reduced sexual desire, our findings could be interpreted as consistent with the previous literature if we consider such an outcome as an indirect indicator of reduced sexual desire; in any case, interpretations should remain cautious.

Despite these findings, fatigue is rarely considered a primary variable in related studies. Instead, it is often hypothesized to interact with other factors, such as depression, breastfeeding, or genital trauma [15–17]. Literature lacks studies that specifically investigate the association between intercourse to satisfy the partner and pain during sex, which is open to multiple interpretations. However, some findings may indirectly support our results, considering our outcome closely associated with a lack of sexual desire and satisfaction in women [1].

Several previous articles indicate a decline in sexual function following childbirth compared to the pre-pregnancy period. Specifically, studies report an increase in perineal pain and dyspareunia, a decrease in sexual desire and arousal capacity, as well as a reduction in the intensity and duration of orgasm during the first three postpartum months, with gradual improvement over the subsequent six months [4, 9, 10, 12, 18, 19]. These findings may be consistent with our observation that early postpartum sex was often motivated by partner satisfaction.

### Limitations

This research is subject to several limitations. Firstly, given the cross-sectional observational design, temporal relationships between exposures and outcomes cannot be established; therefore, the observed associations should not be interpreted as causal and may be influenced by residual confounding. Secondly, the questionnaire was distributed via social media websites, and this may have resulted in a self-selection bias, potentially overrepresenting younger, more digitally literate, and more health-conscious women; hence, the respondents did not represent the general population of pregnant and postpartum women. Third, the inability to assess participation and completion rates due to the anonymous nature of the survey prevented the evaluation of non-response bias. Fourth, while the questionnaire was constructed following a literature review of published papers, it failed to utilize validated psychometric tools (PROMs), thereby limiting comparability and the psychometric strength of assessment; also, the questionnaire reliability and construct validity could not be fully established, and comparisons with studies using standardized tools should be interpreted with caution.

The outcomes were assessed using single dichotomous items. This approach inevitably limits the granularity of the data, as it does not capture the frequency, emotional context, level of distress related to the experience, gradations of desire, motivation, or relational negotiation, thus potentially oversimplifying complex sexual and emotional experiences. However, the binary format was intentionally chosen to enhance clarity and respondent comfort, reduce social desirability bias, and maximize completion rates in an anonymous survey addressing a sensitive and potentially stigmatized topic. Additionally, use of self-reported data may have introduced recall bias or social desirability bias, particularly considering that the subject matter was sensitive. Lastly, the absence of clinical and obstetric data (e.g., mode of delivery, perineal trauma, conditions at breastfeeding) restricted further stratification of answers, which would have enhanced the interpretation of some results; this limitation prevented a more nuanced interpretation of postpartum sexual experiences, which are known to be influenced by delivery-related factors and breastfeeding.

### Implications

This research has significant practice, policy, and research implications. First, the high prevalence of women stating that sexual activity is performed to please their partner – particularly during the postpartum period – elicits insight into potential challenges in sexual autonomy and emotional balance at a formative life stage. This trend, frequently being driven by interpersonal pressure or anticipation of relationship dissolution, warrants explicit recognition by physicians, as it can indicate undermined sexual fulfillment and lack of relational equilibrium.

Second, the high level of correlation between sexual dissatisfaction and problems such as physical fatigue, dyspareunia, and body image disturbance highlight the necessity for a holistic perinatal approach that encompasses body, mind, and relational aspects. Clearly, a need exists for antenatal and postnatal consultations to routinely involve evaluations of sexual health and afford women the forum in which they can voice their concerns and receive individually tailored support.

Third, the low rate of sexual counseling implies an important gap in communication between obstetric care providers and women. This points toward more effective professional training and revised guidelines that explicitly incorporate sexual health as a fundamental aspect of perinatal care. These training programs need to focus both on clinical and communication skills to broach a sensitive but crucial issue. Furthermore, our findings highlight the value of partner engagement. Sexuality during the perinatal period is usually felt with respect to a partner; hence, couple-based counseling or education for partners may be helpful in promoting mutual understanding and joint responsibility.

Our results underscore the imperative for cultural transformation on a larger scale. The views of sexuality during pregnancy as well as the postpartum state remain influenced by stigma, taboos, and gender-based ideals. Researchers should develop and validate questionnaires specifically investigating this aspect of women’s lives, aligning key items with existing patient-reported outcome measures to enhance comparability across studies. Health organizations, education systems, and the media must collaborate in dismantling these deeply ingrained norms, fostering sexual health literacy as a key public health goal. Under the umbrella of a universal healthcare system like the Italian one, the application of these principles within regular care processes could substantially improve the health of women and couples throughout their reproductive life.

## Conclusions

This study sheds light on the complexity of female sexual experience during pregnancy and postpartum, highlighting how relational, physical, and psychological factors interplay in shaping sexual satisfaction. A significant proportion of women reported engaging in sexual activity to satisfy their partner, especially after childbirth, often in the presence of fatigue, dyspareunia, or emotional discomfort. These findings underscore the need to address sexuality as a core component of maternal health. Health professionals should be equipped to explore this domain proactively, offering individualized counseling and support. Promoting open dialogue, validating women’s experiences, and addressing sociocultural pressures that undermine sexual agency are essential steps toward improving perinatal care.

## Supplementary Information

Supplementary Material 1.

## Data Availability

Individual-level data that support the findings of this article are available upon reasonable request from the corresponding author.
