# Supplementary material for: Sexual experiences of women during and after pregnancy: a survey-based study
Source: BMC Pregnancy Childbirth. 2026 Jul 14;26:1129. doi: 10.1186/s12884-026-09648-3 (PMC13647769; doi:10.1186/s12884-026-09648-3)
Supplement: Supplementary file 1 [file 12884_2026_9648_MOESM1_ESM.docx]

**Sexual experiences of women during and after pregnancy: a survey-based study**

**SUPPLEMENTARY MATERIAL**

|  | **1st trimester** | **2nd trimester** | **3rd trimester** | **p-value** |
| --- | --- | --- | --- | --- |
| Number | 63 | 70 | 113 |  |
| Increased libido during search |  |  |  | 0.51 |
| No | 29 (46.0%) | 25 (35.7%) | 45 (39.8%) |  |
| Yes | 34 (54.0%) | 44 (62.9%) | 68 (60.2%) |  |
| Missing | 0 (0.0%) | 1 (1.4%) | 0 (0.0%) |  |
| Increased libido during pregnancy |  |  |  | 0.023 |
| No | 46 (73.0%) | 44 (62.9%) | 59 (52.2%) |  |
| Yes | 17 (27.0%) | 26 (37.1%) | 54 (47.8%) |  |
| Intercourse frequency in pregnancy |  |  |  | 0.12 |
| Increased | 7 (11.1%) | 8 (11.4%) | 19 (16.8%) |  |
| Decreased | 42 (66.7%) | 49 (70.0%) | 84 (74.3%) |  |
| No changes | 14 (22.2%) | 13 (18.6%) | 10 (8.8%) |  |
| Difficult intercourse due to pain |  |  |  | 0.018 |
| No | 46 (73.0%) | 50 (71.4%) | 62 (54.9%) |  |
| Yes | 17 (27.0%) | 20 (28.6%) | 51 (45.1%) |  |
| Fear for penetration |  |  |  | 0.064 |
| No | 25 (39.7%) | 23 (32.9%) | 28 (24.8%) |  |
| A little | 18 (28.6%) | 17 (24.3%) | 47 (41.6%) |  |
| Yes | 20 (31.7%) | 30 (42.9%) | 38 (33.6%) |  |
| Orgasm important |  |  |  | 0.71 |
| No | 2 (3.2%) | 2 (2.9%) | 3 (2.7%) |  |
| A little | 10 (15.9%) | 8 (11.4%) | 10 (8.8%) |  |
| Yes | 50 (79.4%) | 59 (84.3%) | 99 (87.6%) |  |
| Missing | 1 (1.6%) | 1 (1.4%) | 1 (0.9%) |  |
| Conversation on sexual changes |  |  |  | 0.42 |
| No | 25 (39.7%) | 23 (32.9%) | 36 (31.9%) |  |
| Yes, completely | 19 (30.2%) | 15 (21.4%) | 30 (26.5%) |  |
| Yes, partially | 19 (30.2%) | 32 (45.7%) | 46 (40.7%) |  |
| Missing | 0 (0.0%) | 0 (0.0%) | 1 (0.9%) |  |
| Emotional connection with partner |  |  |  | 0.75 |
| Yes | 56 (88.9%) | 61 (87.1%) | 96 (85.0%) |  |
| No / a little | 7 (11.1%) | 9 (12.9%) | 17 (15.0%) |  |
| Partner's support on satisfaction |  |  |  | 0.58 |
| Yes | 52 (82.5%) | 59 (84.3%) | 90 (79.6%) |  |
| No / a little | 9 (14.3%) | 11 (15.7%) | 23 (20.4%) |  |
| Missing | 2 (3.2%) | 0 (0.0%) | 0 (0.0%) |  |
| Sex for man's pleasure |  |  |  | 0.81 |
| No | 28 (44.4%) | 33 (47.1%) | 56 (49.6%) |  |
| Yes | 35 (55.6%) | 37 (52.9%) | 57 (50.4%) |  |
| Physical changes |  |  |  | 0.23 |
| Comfortable with | 25 (39.7%) | 31 (44.3%) | 43 (38.1%) |  |
| Neutral | 25 (39.7%) | 17 (24.3%) | 32 (28.3%) |  |
| Uncomfortable with | 13 (20.6%) | 22 (31.4%) | 38 (33.6%) |  |
| Decreased energy |  |  |  | 0.55 |
| No | 17 (27.0%) | 24 (34.3%) | 31 (27.4%) |  |
| Yes | 46 (73.0%) | 46 (65.7%) | 82 (72.6%) |  |
| Impact of satisfaction on well-being |  |  |  | 0.70 |
| Yes | 31 (49.2%) | 30 (42.9%) | 51 (45.1%) |  |
| No / a little | 31 (49.2%) | 40 (57.1%) | 62 (54.9%) |  |
| Missing | 1 (1.6%) | 0 (0.0%) | 0 (0.0%) |  |
| Fear of jeopardizing relationship |  |  |  | 0.28 |
| Never | 39 (61.9%) | 38 (54.3%) | 69 (61.1%) |  |
| Sometimes | 18 (28.6%) | 28 (40.0%) | 30 (26.5%) |  |
| Often | 6 (9.5%) | 4 (5.7%) | 14 (12.4%) |  |
| Counselling with ObGyn on sex |  |  |  | 0.10 |
| No | 48 (76.2%) | 63 (90.0%) | 92 (81.4%) |  |
| Yes | 15 (23.8%) | 7 (10.0%) | 21 (18.6%) |  |
| Advice from ObGyn on sex |  |  |  | 0.84 |
| No | 51 (81.0%) | 59 (84.3%) | 95 (84.1%) |  |
| Sought | 12 (19.0%) | 11 (15.7%) | 18 (15.9%) |  |
|  |  |  |  |  |

**Table A1.** Women's responses during pregnancy (group differences were evaluated using the chi-squared test, with p-values < 0.05 indicating statistical significance).

Approximately 60% of women reported increased libido while seeking pregnancy, with no differences across response times. There was a gradual and significant increase in woman-reported libido from the first to the third trimester; 25-30% percent of women had intercourse as frequently or more often than before pregnancy, while 70% less frequently. Women who reported pain during intercourse were about 28% in the first/second trimester but were 45% in the third trimester. Fear of vaginal penetration was reported by 60% of women in the first trimester and 75% in the third trimester. More than 95% of the women believed that orgasm was important for couple's sex life. During pregnancy, 30-40% of women had never had a conversation with their partner about changes in sexuality, although 85-90% of women reported being emotionally connected with the partner. Support from the partner on sexual satisfaction was received by 80% of women, but 50-55% reported that they had sex only for male pleasure. Only 20% of women felt uncomfortable with their physical changes in the first trimester while 34% in the third trimester. Women who reported reduced energy were 73% in the first and third trimesters, and 66% in the second one, with no significant differences. A significant impact of sexual satisfaction on couple well-being was reported by 45% of women, and about 40% declared that they were sometimes or often afraid of jeopardizing the relationship. As in the total population, only 18% had a conversation with their gynecologist about sexuality, while only 17% sought advice.

|  | **<2 mo postpartum** | **2-6 mo postpartum** | **p-value** |
| --- | --- | --- | --- |
| Number | 49 | 334 |  |
| Increased pleasure |  |  | 0.61 |
| No | 39 (79.6%) | 285 (85.3%) |  |
| Yes | 5 (10.2%) | 47 (14.1%) |  |
| Missing | 5 (10.2%) | 2 (0.6%) |  |
| Childbirth’s influence on coupe |  |  | 0.47 |
| Enough | 12 (24.5%) | 88 (26.3%) |  |
| A lot | 10 (20.4%) | 96 (28.7%) |  |
| Not at all | 14 (28.6%) | 68 (20.4%) |  |
| A little | 13 (26.5%) | 82 (24.6%) |  |
| Sex postpartum within 2 months |  |  | 0.49 |
| Not yet | 24 (49.0%) | 146 (43.7%) |  |
| Yes | 25 (51.0%) | 188 (56.3%) |  |
| Intercourse frequency vs before pregnancy |  |  | 0.001 |
| Increased | 1 (2.0%) | 21 (6.3%) |  |
| Decreased | 26 (53.1%) | 256 (76.6%) |  |
| Not changed | 18 (36.7%) | 57 (17.1%) |  |
| Missing | 4 (8.2%) | 0 (0.0%) |  |
| Intercourse frequency vs during pregnancy |  |  | 0.024 |
| Increased | 7 (14.3%) | 89 (26.6%) |  |
| Decreased | 19 (38.8%) | 153 (45.8%) |  |
| Not changed | 22 (44.9%) | 92 (27.5%) |  |
| Missing | 1 (2.0%) | 0 (0.0%) |  |
| Difficult sex after delivery |  |  | 0.21 |
| No | 20 (40.8%) | 122 (36.5%) |  |
| Yes | 23 (46.9%) | 210 (62.9%) |  |
| Missing | 6 (12.2%) | 2 (0.6%) |  |
| Time for sex despite children |  |  | 0.061 |
| Yes | 8 (16.3%) | 97 (29.0%) |  |
| No / a little | 41 (83.7%) | 236 (70.7%) |  |
|  | 0 (0.0%) | 1 (0.3%) |  |
|  |  |  |  |

**Table A2.** Women's responses postpartum (group differences were evaluated using the chi-squared test, with p-values < 0.05 indicating statistical significance).

Only 10-14% of women reported that sexual pleasure had increased after delivery. Childbirth had an influence on the relationship in 50% of women. Women who had sexual intercourse within two months of childbirth were about half. Compared to the preconception period, the frequency of sexual intercourse decreased in 53% of women within 2 months postpartum and in 77% of women within 6 months postpartum. In contrast, compared with pregnancy, frequency decreased in 39% and 46% of cases, respectively. Difficulty in having sexual intercourse increased from 47% to 63% from 2 months to 6 months postpartum, although not significantly. In the first 2 months postpartum, only 16% of women reported having time for sexual intercourse despite having children, a percentage that increased (though not significantly) to 29% by 6 months postpartum.

| **Pregnancy sub-population** (n=241) | | | |
| --- | --- | --- | --- |
| Difficulty or pain during sex | **OR** | **p-value** | **95% CI** |
| 2nd vs 1st trimester | 1,3 | 0,515 | 0,5 to 3,3 |
| 3rd vs 1st trimester | 3,0 | 0,007 | 1,3 to 6,6 |
|  |  |  |  |
| 30-34 vs <30 | 0,6 | 0,249 | 0,3 to 1,4 |
| 35-39 vs <30 | 0,4 | 0,078 | 0,2 to 1,1 |
| >39 vs <30 | 0,2 | 0,131 | 0 to 1,7 |
|  |  |  |  |
| Multiparous vs primigravida | 0,7 | 0,408 | 0,4 to 1,5 |
|  |  |  |  |
| Sex for man's pleasure vs not | 2,1 | 0,018 | 1,1 to 3,9 |
|  |  |  |  |
| Decreased energy vs not | 1,4 | 0,312 | 0,7 to 2,9 |
|  |  |  |  |
| Counselling with ObGyn on sex vs not | 1,6 | 0,270 | 0,7 to 3,7 |
|  |  |  |  |
| Increased libido while seeking pregnancy | 1,4 | 0,287 | 0,7 to 2,7 |
|  |  |  |  |
| Decreased frequency of sex in pregnancy vs not | 1,8 | 0,324 | 0,6 to 5,9 |
|  | 2,0 | 0,330 | 0,5 to 7,5 |
|  |  |  |  |
| Increased libido during pregnancy | 0,6 | 0,203 | 0,3 to 1,3 |
|  |  |  |  |
| Little vs no fear for penetration | 0,6 | 0,157 | 0,2 to 1,3 |
| Great vs no fear for penetration | 1,2 | 0,654 | 0,5 to 2,7 |
|  |  |  |  |
| Little vs no importance of orgasm | 0,9 | 0,931 | 0,1 to 7,2 |
| Great vs no importance of orgasm | 0,6 | 0,637 | 0,1 to 4,3 |
|  |  |  |  |
| Complete vs no conversation with partner on sex | 1,4 | 0,404 | 0,6 to 3,3 |
| Partial vs no conversation with partner on sex | 1,7 | 0,144 | 0,8 to 3,6 |
|  |  |  |  |
| No / poor emotional connection with partner vs yes | 1,3 | 0,540 | 0,5 to 3,4 |
|  |  |  |  |
| Neutral vs comfortable with physical changes in pregnancy | 2,5 | 0,019 | 1,2 to 5,2 |
| Uncomfortable vs comfortable with physical changes in pregnancy | 1,9 | 0,114 | 0,9 to 4 |
|  |  |  |  |
| No / poor impact of sexual satisfaction on couple vs yes | 1,1 | 0,694 | 0,6 to 2,2 |
|  |  |  |  |
| Sometimes vs never fear for jeopardizing relationship | 0,6 | 0,156 | 0,3 to 1,2 |
| Always vs never fear for jeopardizing relationship | 1,0 | 0,997 | 0,3 to 3 |
| OR: Odds Ratio; CI: confidence interval; ObGyn: Obstetrician-Gynecologist | | | |

**Table A3.** Logistic regression model for the secondary outcome in the pregnancy sub-population

| **Postpartum sub-population** (n=383) | | | |
| --- | --- | --- | --- |
| Sex within two months | **OR** | **p-value** | **95% CI** |
| 2-6 vs <2 months postpartum | 1,1 | 0,9 | 0,5 to 2,2 |
|  |  |  |  |
| 30-34 vs <30 | 1,2 | 0,6 | 0,6 to 2,3 |
| 35-39 vs <30 | 1,1 | 0,7 | 0,6 to 2,4 |
| >39 vs <30 | 0,8 | 0,7 | 0,3 to 2,2 |
|  |  |  |  |
| Multiparous vs primigravida | 1,0 | 0,9 | 0,6 to 1,6 |
|  |  |  |  |
| Sex for man's pleasure vs not | 1,8 | 0,0 | 1,1 to 3,1 |
|  |  |  |  |
| Decreased energy vs not | 0,7 | 0,3 | 0,4 to 1,4 |
|  |  |  |  |
| Counselling with ObGyn on sex vs not | 1,1 | 0,7 | 0,6 to 2,1 |
|  |  |  |  |
| Great vs moderate influence of childbirth on couple | 0,5 | 0,0 | 0,3 to 1 |
| No vs moderate influence of childbirth on couple | 2,9 | 0,0 | 1,4 to 6 |
| Little vs moderate influence of childbirth on couple | 2,7 | 0,0 | 1,4 to 5,3 |
|  |  |  |  |
| Increased sexual pleasure postpartum vs not | 0,6 | 0,2 | 0,3 to 1,2 |
|  |  |  |  |
| Decreased vs increased frequency of sex before pregnancy | 0,8 | 0,7 | 0,3 to 2,7 |
| Not changed vs increased frequency of sex before pregnancy | 2,1 | 0,2 | 0,6 to 7,3 |
|  |  |  |  |
| Decreased vs increased frequency of sex in pregnancy | 1,4 | 0,3 | 0,7 to 2,7 |
| Not changed vs increased frequency of sex in pregnancy | 0,9 | 0,9 | 0,5 to 1,9 |
|  |  |  |  |
| Difficult sex postpartum vs not | 0,9 | 0,7 | 0,5 to 1,5 |
|  |  |  |  |
| No / little time for sex despite children vs a lot | 0,4 | 0,0 | 0,2 to 0,7 |
|  |  |  |  |

**Table A4.** Logistic regression model for the secondary outcome in the postpartum sub-population

**Appendix A1.** Sexual Satisfaction Questionnaire for Pregnant Women

Age ____

Number of pregnancies ___

Weeks of current pregnancy ___

During the period of trying to conceive, how much did your libido increase?

- Very much
- Fairly
- A little
- Not at all

Have you noticed changes in your libido (sexual desire) during pregnancy?

- Increased
- Decreased
- No change

If yes, in which trimester of pregnancy did you notice the greatest change?

- First (up to 13 weeks)
- Second
- Third

Has the frequency of your sexual intercourse changed during pregnancy?

- Increased
- Decreased
- No change

If yes, in which trimester did you notice the greatest change?

- First
- Second
- Third

Have you experienced difficulty or pain during sexual intercourse in pregnancy?

- Yes
- No

If yes, what type of difficulty or pain did you experience?

- Pain during penetration
- Pelvic pain
- Vaginal dryness
- Other ______________

Have you ever used sex toys in your sexual life?

- Always
- Often
- Sometimes
- Never

If yes:
During pregnancy, did you continue to use them?

- More than before
- As before
- Less than before
- No

Has pregnancy caused any change in your couple’s sexual practices?

- No change
- I avoided complete vaginal penetration but maintained oral or other types of intercourse.
- I preferred external stimulation, possibly with the help of sex toys.
- I avoided any kind of sexual activity during pregnancy.

If yes, in which trimester did you notice the greatest change?

- First
- Second
- Third

Did complete vaginal intercourse cause you fear or concern during pregnancy?

- Very much
- Fairly
- A little
- Not at all

If present, how much did this concern affect the well-being and sexual satisfaction of the couple?

- Very much
- Fairly
- A little
- Not at all

What is your main concern about having penetrative intercourse during pregnancy?

- Fear of miscarriage in the first trimester
- Fear of harming the fetus in the second or third trimester
- Fear of preterm birth
- Fear of rupture of the amniotic membranes
- Risk of infections that could harm the fetus
- Other ____________________

Has the achievement of orgasm and pleasure changed during pregnancy?

- Increased
- Decreased
- No change

If yes, in which trimester did you notice the greatest change?

- First
- Second
- Third

During pregnancy, how important is reaching orgasm?

- Very
- Fairly
- A little
- Not at all

In which position do you most easily reach orgasm during pregnancy?

________________________________________________________________________

How would you describe your overall sexual satisfaction during this pregnancy?

- Very satisfied
- Satisfied
- Neither satisfied nor dissatisfied
- Dissatisfied
- Very dissatisfied

How emotionally connected do you feel to your partner during pregnancy?

- Very
- Fairly
- A little
- Not at all

Have you discussed with your partner the changes in your sexual life during pregnancy?

- Yes, completely
- Yes, partially
- No

Do you feel supported by your partner regarding your sexual satisfaction during pregnancy?

- Very much
- Fairly
- A little
- Not supported

Have you ever had intercourse with your partner solely to satisfy his pleasure?

- Always
- Often
- Sometimes
- Never

Have you ever felt obligated to have sexual intercourse with your partner during pregnancy to avoid compromising the relationship?

- Always
- Often
- Sometimes
- Never

Do you feel comfortable with your body changes during pregnancy in relation to your sexual life?

- Very comfortable
- Comfortable
- Neutral
- Uncomfortable
- Very uncomfortable

Have you experienced changes in your energy level that affected your sexual life?

- Yes, increased energy
- Yes, decreased energy
- No change

Do you feel that your sexual satisfaction can influence your well-being during pregnancy?

- Very much
- Fairly
- A little
- Not at all

Have you ever been afraid of compromising your relationship with your partner because of your sexuality during pregnancy?

- Always
- Often
- Sometimes
- Never

Have you and your partner ever thought about seeing a sexologist during pregnancy?

- Always
- Often
- Sometimes
- Never

Have you ever had a couple session with a sexologist (different from your gynecologist) to address sexual issues during pregnancy?

- Yes, completely
- Yes, partially
- No

Have you talked to your gynecologist about sexual issues encountered during pregnancy?

- Yes, completely
- Yes, partially
- No

If yes, what did you ask them?

________________________________________________________________________

If yes, did you feel understood, listened to, and satisfied with the answers received?

- Very
- Fairly
- A little
- Not at all

If no, why?

- Because I have no sexual problems with my partner
- Because I am ashamed to talk about the topic
- Because I am afraid it might seem like a whim
- Because I have no sexual desire during pregnancy and I’m fine with it

Have you ever asked your gynecologist for advice about sexuality with your partner during pregnancy?

- Yes
- No

Did the couple session with the gynecologist satisfy and help you in dealing with issues that emerged with your partner?

- Very
- Fairly
- A little
- Not at all

If yes, did you feel understood, listened to, and satisfied with the answers received?

- Very
- Fairly
- A little
- Not at all

If you had a gynecologist or sexologist expert in the field in front of you, what would you sincerely ask them?

________________________________________________________________________

Section for women who have already had a previous childbirth

Has the experience of childbirth influenced your couple’s sexual life?

- Very much
- Fairly
- A little
- Not at all

Has your partner’s participation in the childbirth (if applicable) influenced the couple’s sexual desire?

- Yes, it increased desire
- No, childbirth reduced desire
- It remained the same
- He did not attend

After how long did you and your partner resume sexual activity after childbirth?

- One week
- Two weeks
- One month
- Three months
- More than six months

After your child was born, were you and your partner able to find time to satisfy your sexual desire?

- Very much
- Fairly
- A little
- Not at all
